# Characterization of the F-box Proteins FBXW2 and FBXL14 in the Initiation of Bone Regeneration in Transplants given to Nude Mice

**DOI:** 10.2174/1874120701812010075

**Published:** 2018-10-18

**Authors:** Mari Akiyama

**Affiliations:** Department of Biomaterials, Osaka Dental University, Osaka 573-1121, Japan

**Keywords:** Cultured bovine-periosteum-derived cells, FBXW2, FBXL14, Bone regeneration, Osteocalcin, TRAP staining

## Abstract

**Background:**

Cultured bovine-periosteum-derived cells can form three-dimensional structures on tissue culture dishes without artificial scaffolding material, can induce bone regeneration *in vivo*. The utility of cultured bovine-periosteum-derived cells for bone tissue regeneration after their transplantation into nude mice has been reported, the precise F-box molecular mechanism was unclear.

**Objective:**

The aim of this study was to investigate the specific F-box proteins required for bone regeneration by cultured bovine-periosteum-derived cells *in vitro*.

**Methods:**

In the present study, periosteum tissue and cultured periosteum-derived cells were cultured for 5 weeks *in vitro* and then embedded in collagen gel with a green tissue-marking dye. Electrophoresis and immunohistochemistry were used to identify the specific F-box proteins required for tissue bone regeneration.

**Results:**

The bovine-periosteum-derived cells were observed to form bone shortly after the expression of F-box proteins. After the initial phase of bone formation, the expression of the F-box proteins ceased. FBXW2 was shown to be expressed in the periosteum, but not in cultured periosteum-derived cells. Furthermore, FBXL14 disappeared during bone formation.

**Conclusions:**

Bone regeneration requires progenitor cells, such as bovine-periosteum-derived cells and the activation of the F-box Proteins FBXW2 and FBXL14, over time the expression of these proteins ceases. Further scientific and clinical trials are needed to investigate how the F-box Proteins can be used therapeutically to treat osteoporosis and osteonecrosis.

## 1 INTRODUCTION

The periosteum is a membrane that surrounds bones and is important in bone regeneration [1, 2]. It consists of a fibrous outer layer and an inner cambium layer that continuously generates osteoblasts, causing bone growth [3]. The periosteum contains osteoblasts, fibroblasts that are indistinguishable from the adjacent osteoblasts, multipotent cells that can differentiate into osteoblasts, and chondrocyte [3-5]. The periosteum has a different ultrastructure and osteogenic potential according to its location [6]. For instance, the calvarial periosteum has less osteogenic potential than the tibial periosteum, as described by Dwek [3]. The osteoblastic differentiation and bone regeneration potential of periosteum-derived cells, which are a mixture of multipotent cells and fibroblasts, have been studied [6-9]. For example, cultured bovine-periosteum-derived cells can form three-dimensional multiple-cell-layered structures on tissue culture dishes without artificial scaffolding material, and can subsequently be used for bone regeneration *in vivo* [10, 11]. Despite advances in the use of cultured bovine-periosteum-derived cells for bone tissue regeneration after their transplantation into nude mice, the precise mechanism of bone formation remains unclear. Specifically, it is unclear which proteins are required to form the multicellular layer that is necessary to initiate bone regeneration.

Previous studies have identified some of the proteins that are expressed by cultured periosteum-derived cells. For example, using Mass Spectrometry (MS) and Immunohistochemistry (IHC), Akiyama [12, 13] recently reported that uveal autoantigen with coiled-coil domains and ankyrin repeats (UACA), exosome complex component RRP45 (EXOSC9), thioredoxin-related transmembrane protein 2 (TMX2), beta-tubulin, and F-box/leucine-rich repeat protein 14 (FBXL14) are expressed in cultured bovine-periosteum-derived cells. Another study identified candidate proteins present in cultured bovine-periosteum-derived cells using MS. However, only small peptide fragments were detected, making their identification difficult. Some of the proteins were confirmed with IHC in paraffin sections of cultured bovine-periosteum-derived cells using antibodies directed against each candidate protein [12, 13]. However, some proteins in these cells could not be confirmed [12].

One of the candidate proteins detected with MS that is not expressed in the multilayer of cultured bovine-periosteum-derived cells is the F-box protein F-box/WD repeat-containing protein 2 (FBXW2). However, the F-box protein FBXL14 was identified with IHC. The F-box proteins are divided into three classes: FBXW, defined by a WD repeat motif; FBXL, defined by a leucine-rich repeat motif; and FBXO, which includes proteins with an F-box and either another or no other binding motif [14]. F-box proteins recognize E3 ubiquitin ligases, but other roles, beyond ubiquitination, have also recently been reported [15-17]. FBXW11, FBXL1, and FBXW7 are the three best-characterized of the 69 F-box proteins [18, 19] and FBXW7 is particularly well characterized in tumors [17]. FBXW2 is reported to regulate placental cell migration and invasion [20, 21], but its roles in the periosteum and bone regeneration are still unclear. In a previous study, the original periosteum was removed from primary cultured cells and only the bulk of the cultured periosteum-derived cells was investigated [12, 13]. Cultured periosteum-derived cells and periosteum were examined in parallel in that study, and FBXW2 was found to be expressed in the periosteum but not in the cultured periosteum-derived cells.

Several more factors present in the periosteum have been identified. For example, a recent review by Lin *et al*. [22] described the interactions between bone morphogenetic protein, fibroblast growth factor, Hedgehog, Notch, platelet-derived growth factor, WNT, and inflammation signaling during bone regeneration [22]. Cherry *et al*. [23] also demonstrated that the periosteum displays properties of mesenchymal stromal/stem cells, although equivalent cells were not identified in the periosteum because no specific markers were available. Two osteogenic markers, osteocalcin (for osteoblasts) and Tartrate-Resistant Acid Phosphatase (TRAP; for osteoclasts), are known [24, 25]. Although significant research into the proteins related to bone formation, including osteocalcin and TRAP, has been conducted, the interaction between FBXW2 and the periosteum has not been investigated. Therefore, the aim of this study was to investigate the specific F-box proteins required for bone regeneration by cultured bovine-periosteum-derived cells *in vitro*.

## 2 MATERIALS AND METHODS

### 2.1 Cell Culture

All animal experiments were performed according to the guidelines for animal experimentation of Osaka Dental University (no. 15-01007). Bovine periosteum from the legs of Japanese black cattle was used for explant culture for 5 weeks in medium 199 containing ascorbic acid, as described previously [10]. The Japanese black cattle were 30-month-old adult steers (ex-male) or cows obtained from a slaughterhouse (Kobe Chuo Chikusan, Kobe, Japan) preparing meat products. Twenty-four hours after the death of the animal, the bovine periosteum was used for cell culture, according to the guidelines for animal experimentation at Osaka Dental University (no. 15-01007). Two pieces of periosteum tissue (5 × 5 mm^2^) were placed on 100-mm dishes for 5 weeks. It has previously been reported that bone was successfully regenerated in culture medium containing ascorbic acid, whereas medium without ascorbic acid bone did not allow this regeneration [11]. Therefore, in this study, all experiments were performed with ascorbic acid, and the culture medium was changed once a week. For MS, the medium was changed to medium without Fetal Bovine Serum (FBS) after 4 weeks, and the cells were cultured for 1 week in the FBS-free medium. The supernatants without FBS were collected.

### 2.2 MS and Selection of FBXW2

As in previous studies, the supernatant samples from cultured periosteum-derived cells were subjected to two-dimensional electrophoresis [12, 13]. When the cells were cultured in the presence of ascorbic acid, spots containing type 1 collagen and noncollagenous proteins were observed, and those spots were analyzed with MS/MS using *Micro TOF-Q* mass spectrometer (BrukerDaltonics, Bremen, Germany) [12, 13]. Details of the search parameters and the results of a MASCOT search [26] are shown in Table **1**. As shown in Fig. (**1** and Table **1**), FBXW2 was identified as a candidate protein in this study.

### 2.3 Cross-sections of Periosteum and Cultured Periosteum-derived Cells

The methods used to produce cross-sections of the periosteum and cultured periosteum-derived cells are described in Fig. (**2**). After 5 weeks, both the periosteum and cultured periosteum-derived cells were removed from the tissue culture dishes and embedded in collagen gel (*Cellmatrix Type1-A*, Nitta Gelatin, Osaka, Japan), marked with a green tissue marker (*Tissue Marking Dye*, Falma Co. Ltd, Tokyo, Japan) on top, which indicated the upper sides of the culture dishes. These cell mixtures were fixed in 4% paraformaldehyde (PFA), and the paraffin blocks were sectioned to 6 µm. The paraffin sections were then prepared for IHC.

### 2.4 Immunohistochemistry

In previous studies, peptide fragments of UACA, EXOSC9, TMX2, beta-tubulin, and FBXL14 were detected, with protein sequence coverages of approximately 1%, 3%, 3%, 2%, and 3% for each protein, respectively [12, 13]. Therefore, each protein was confirmed with IHC, as described in the previous studies [12, 13].

The paraffin sections were pretreated with proteinase K (Dako Cytomation, Glostrup. Denmark) and a peroxidase blocking solution (Dako). Two primary antibodies directed against F-box proteins and an antibody directed against osteocalcin were used: anti-FbxW2 antibody (ab5309; Abcam, Cambridge, UK), anti-FBXL14 antibody (SAB2103691-50UG, Sigma-Aldrich Co., St. Louis, MO), and anti-osteocalcin (code no. M042, clone no. OCG2; Takara Bio Inc., Otsu, Japan). Three secondary antibodies were used: horseradish peroxidase (HRP)-labeled anti-goat immunoglobulin (P0449, Dako) for the anti-FbxW2 antibody; Envision+ System HRP-labeled polymer anti-rabbit immunoglobulin (Dako) for the anti-FBXL14 antibody; and the Envision+ System HRP-labeled polymer anti-mouse immunoglobulin (Dako) for the anti-osteocalcin antibody. The anti-FbxW2 antibody was diluted 1:100 and applied to the sections for 2 h; the anti-FBXL14 antibody was diluted to 0.33 μg/ml and applied for 1 h; and the anti-osteocalcin antibody was diluted 1:500 and applied for 4 h. Anti-goat immunoglobulin–HRP was diluted to 1:100 and the Envision solutions were not diluted. Hematoxylin was used to counterstain the nuclei. Independent experiments were repeated three times.

### 2.5 Cell Transplantation and Bone Regeneration

Prior to surgery the animals were given general anesthesia and their vital signs were monitored. The cell transplantation process is shown in Fig. (**1**). which is similar to previous studies [10, 11]. After 5 weeks in culture *in vitro*, the periosteum-derived cells was excised and transplanted into the back skin of three male nude KSN mice (6 weeks old; Shimizu Laboratory Supplies Co., Ltd). Cultured periosteum-derived cells were separated from six pieces of periosteum. Cells embedded in extracellular matrix, such as collagen fibers, were not trypsinized or counted. After 4 weeks, the mice were euthanized with an overdose of isoflurane. The skin from the nude mice and the new bone tissue from the cultured periosteum-derived cells were fixed in 4% PFA. Paraffin sections were prepared for IHC and Hematoxylin and Eosin (HE), von Kossa, and TRAP staining.

## 3 RESULTS

Immunohistochemistry (IHC) was used to determine whether the F-box proteins FBWX2 and FBXL14 were expressed in cultured periosteum-derived cells. Table **2** summarizes the results of IHC in this study. FBXW2 was expressed in the periosteum, whereas FBXL14 was expressed in the cultured periosteum-derived cells, and osteocalcin was expressed in both the cultured periosteum-derived cells and the transplanted cells.

**Fig. (3):**
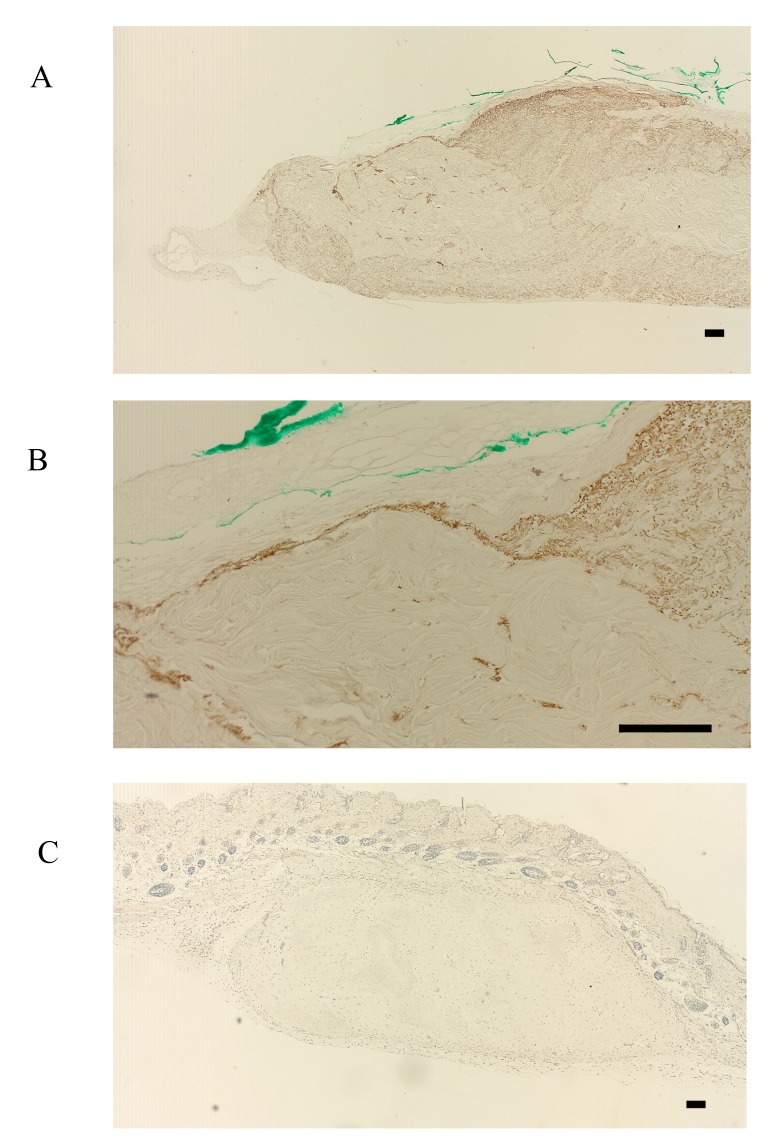
IHC of FBXW2. (**A**) Five weeks after explant culture of bovine periosteum. (**B**) High-power field of (**A**). (**C**) After cell transplantation into nude mouse skin and the formation of new bone tissue. Scale bars: 100 µm.

FBXW2 was expressed in the periosteum, but not in the cultured periosteum-derived cells or transplanted cells (Fig. **3A**-**C**). Although FBXW2 was identified as a putative component of proteins secreted from the cultured periosteum-derived cells with MS, IHC was unable to confirm this finding in previous studies, presumably because FBXW2 was only present in the periosteum (Fig. **3A**, **B**). Consistent with this, the transplanted cells were also negative for FBXW2 (Fig. **3C**).

**Fig. (4):**
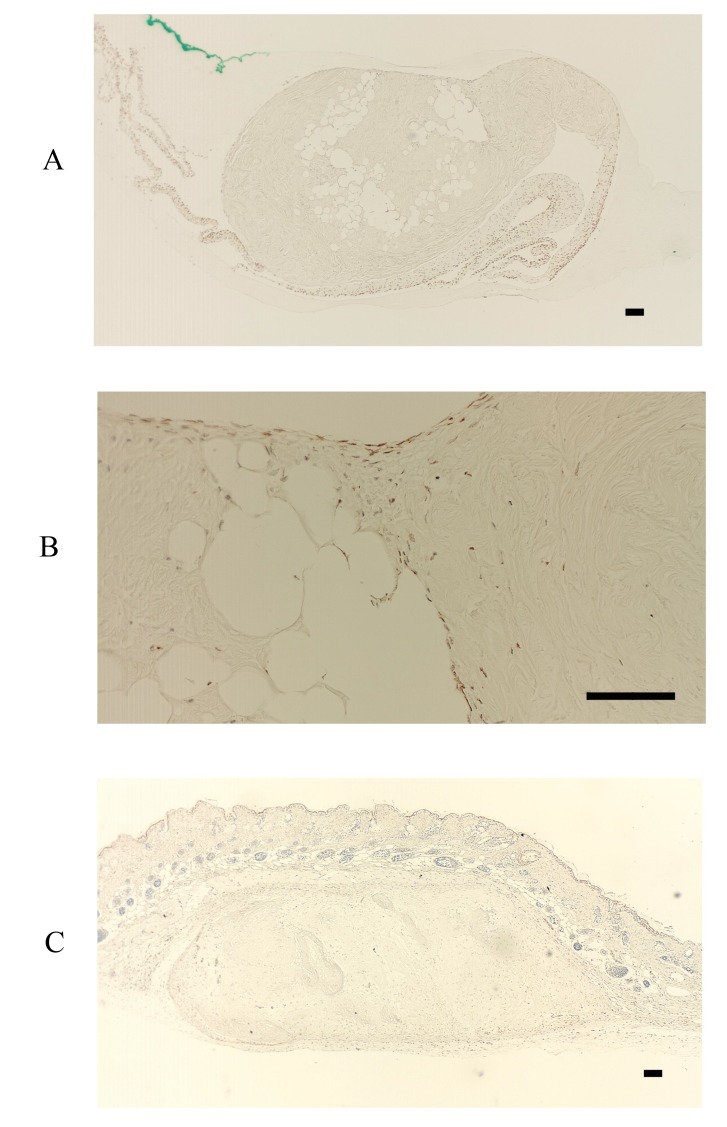
IHC of FBXL14. (**A**) Five weeks after explant culture of bovine-periosteum-derived cells. (**B**) High-power field of (**A**). (**C**) After cell transplantation into nude mouse skin and the formation of new bone tissue. Scale bars: 100 µm.

In contrast, FBXL14 was not expressed in the periosteum, but in the periosteal cells located within the periosteum and in the cultured periosteum-derived cells (Fig. **4A**, **B**). Surprisingly, although FBXL14 was present in the cultured periosteum-derived cells before transplantation, the transplanted cells were negative for FBXL14 (Fig. **4C**). *In vivo*, that part of the skin surface of the nude mice was positive for FBXL14 (Fig. **4C**).

**Fig. (5):**
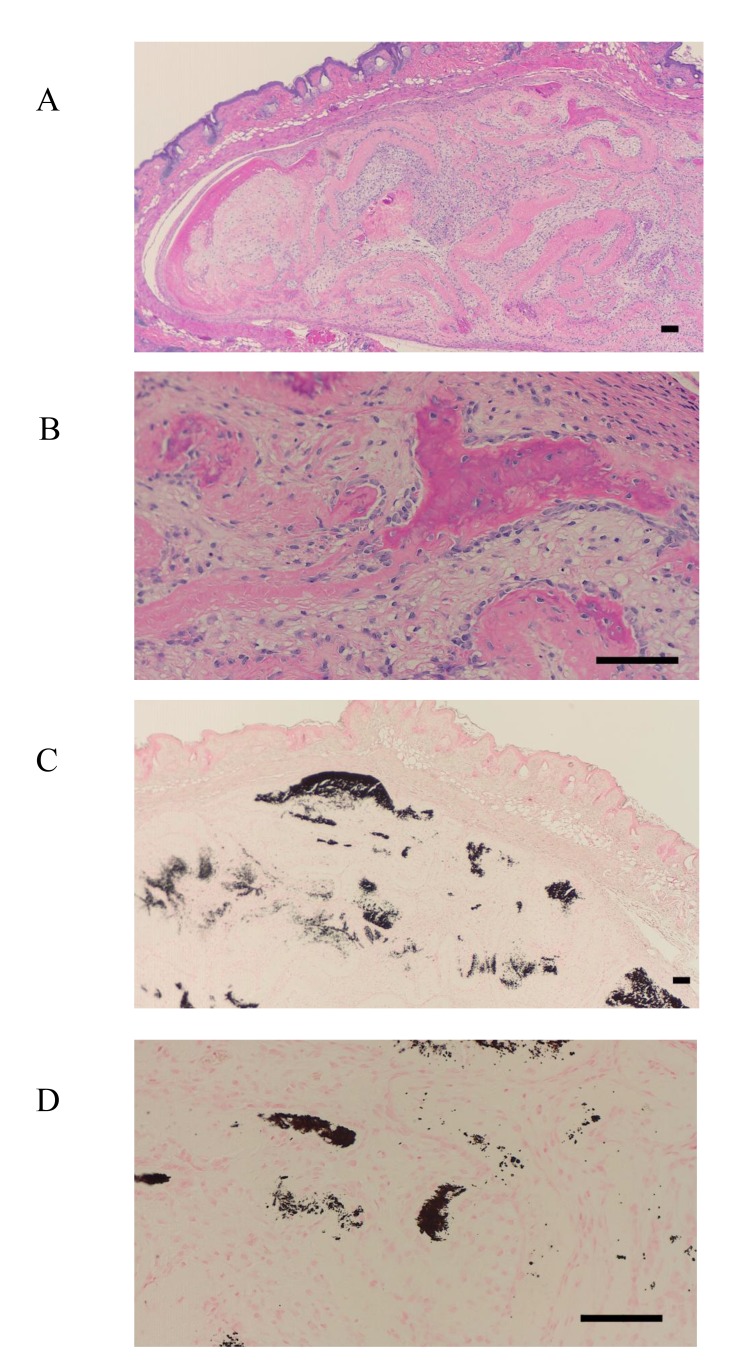
Transplanted bovine-periosteum-derived cells. (**A**) HE staining. (**B**) High-power field of (**A**). (**C**) von Kossa staining. (**D**) High-power field of (**C**). Scale bars: 100 µm.

To localize the F-box proteins FBXW2 and FBXL14 during the process of new bone formation, sections were analyzed 4 weeks after transplantation with HE staining, von Kossa staining, the immunohistochemical detection of osteocalcin, and TRAP staining. It has previously been reported that posttransplantation bone regeneration is completed within 6 weeks [10]. Consistent with this, there was evidence of bone formation at 4 weeks in this study, apparent as partially calcified new bone tissue (Fig. **5A**-**D**). However, at this stage, it was clear that the expression of FBXW2 and FBXL14 had ceased during bone development.

**Fig. (6):**
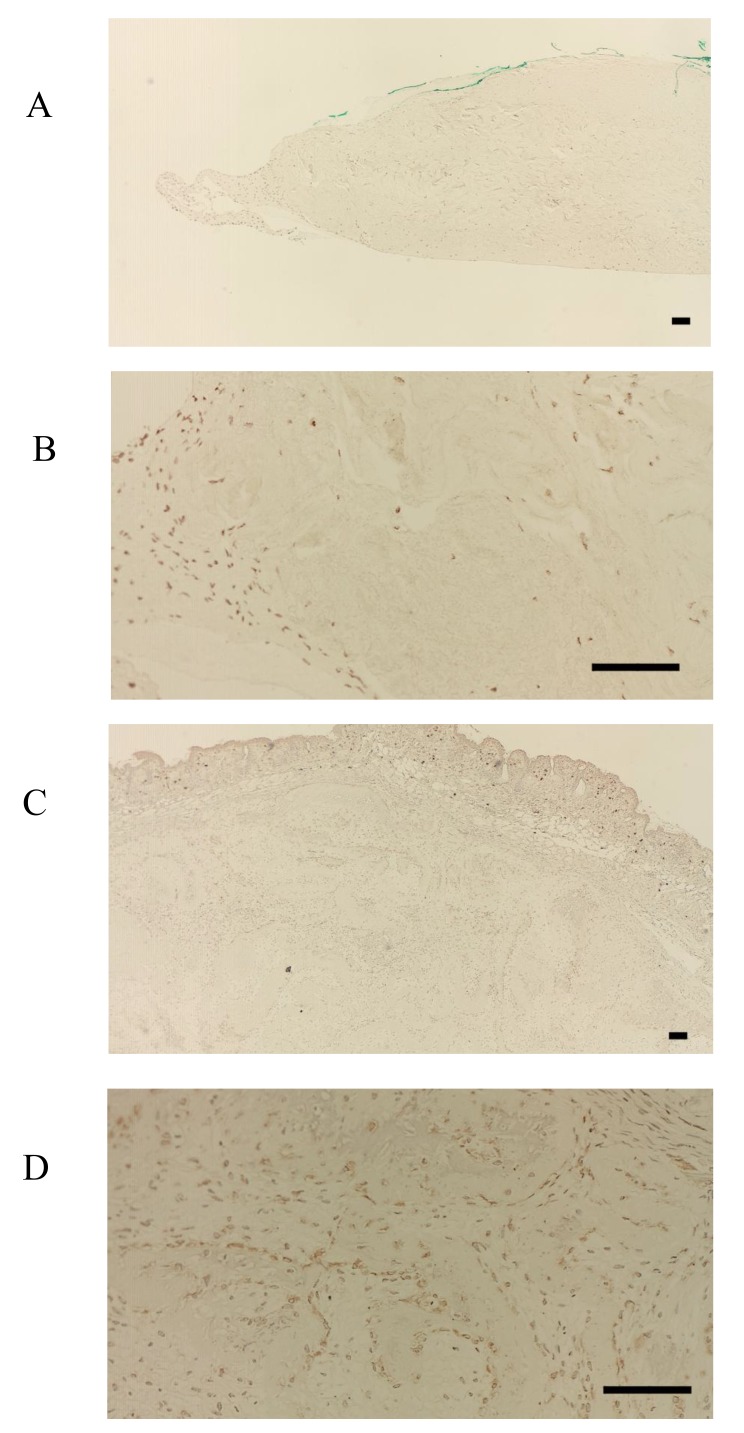
IHC of osteocalcin. (**A**) Five weeks after explant culture of bovine-periosteum-derived cells. (**B**) High-power field of (**A**). (**C**) After cell transplantation into nude mouse skin and the formation of new bone tissue. (**D**) High-power field of (**C**). Scale bars: 100 µm.

**Fig. (7):**
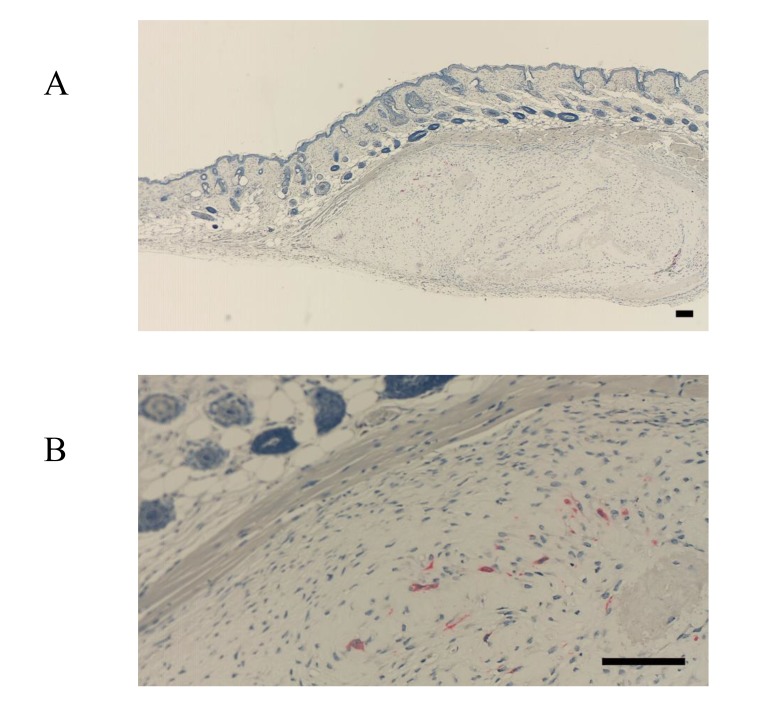
TRAP staining. (**A**) After cell transplantation into nude mouse skin and the formation of new bone tissue. (**B**) High-power field of (**A**). Scale bars: 100 µm.

Osteocalcin was also present in the cultured bovine-periosteum-derived cells and transplanted cells (Fig. **6A**-**D**). However, only one side of the periosteum-derived cell layer was positive for FBXL14, whereas both sides of cell layer were positive for osteocalcin (Fig. **6A**, **B**). Specifically, at 4 weeks posttransplantation, cultured periosteum-derived cells expressing osteocalcin were observed around the new bone tissue and future bone tissue (Fig. **6C**, **D**). In the HE-stained samples in Fig. (**5A**, **B**), the new bone tissue stained strongly pink and osteoblasts were present around the bone tissue. Therefore, the osteocalcin-positive cells in Fig. (**6C**, **D**) appear to be osteoblasts. The results shown in Fig. (**6C**, **D**) also indicate that the cultured bovine-periosteum-derived cells were positive for osteocalcin before transplantation (Fig. **6A**, **B**). Areas near the new bone tissue were also positive for TRAP staining, a marker of osteoclasts (Fig. **7A**, **B**).

## 4 DISCUSSION

The results of this present study are the first to demonstrate that the F-box Proteins FBXW2 and FBXL14 are involved in the initiation of bone regeneration. The results offer the potential to use F-box proteins therapeutically to regenerate bone where bone loss has occurred through diseases involving macrophage activity such as osteoporosis and osteonectrosis, and as coatings on orthopedic and tooth implants to promote healing.

However, the F-box proteins have not been reported to be associated with the periosteum or periosteum-derived cells. The present results explain this phenomenon by showing that the F-box proteins, such as FBXW2 and FBXL14, are present during the initiation phase of cell layer development, but are then lost during bone regeneration.

Recently, using MS and a MASCOT analysis, Akiyama [13] reported that one of the F-box proteins, FBXL14, is present in cultured bovine-periosteum-derived cells. However, an antibody directed against another candidate protein, FBXW2, did not react, or reacted extremely weakly, with bulk sections of cultured bovine-periosteum-derived cells lacking periosteum. This indicates that FBXW2 is present in the periosteum and FBXL14 is present in cultured periosteum-derived cells.

In previous studies, some candidate proteins identified with MS were not detected in subsequent confirmatory experiments, which suggests that those proteins are also only present in the periosteum. Mass spectrometry is an imperfect technique, and sometimes detects false-positive proteins. Therefore, Akiyama [12] combined MS with IHC in that study. In the present study, MS identified proteins not only in the cultured periosteum-derived cells, but also in the periosteum. The utility and importance of protein detection with MS, and its confirmation with IHC, are highlighted in this study. Alternatively, western blotting analyses of the culture supernatants of cultured bovine-periosteum-derived cells have previously been used as an effective validation tool. However, western blotting requires a large volume of protein and fragments of the proteins in the supernatant cannot be detected. In contrast, IHC can detect small volumes of protein with an antibody and can be used to localize the proteins [26].

**Fig. (8):**
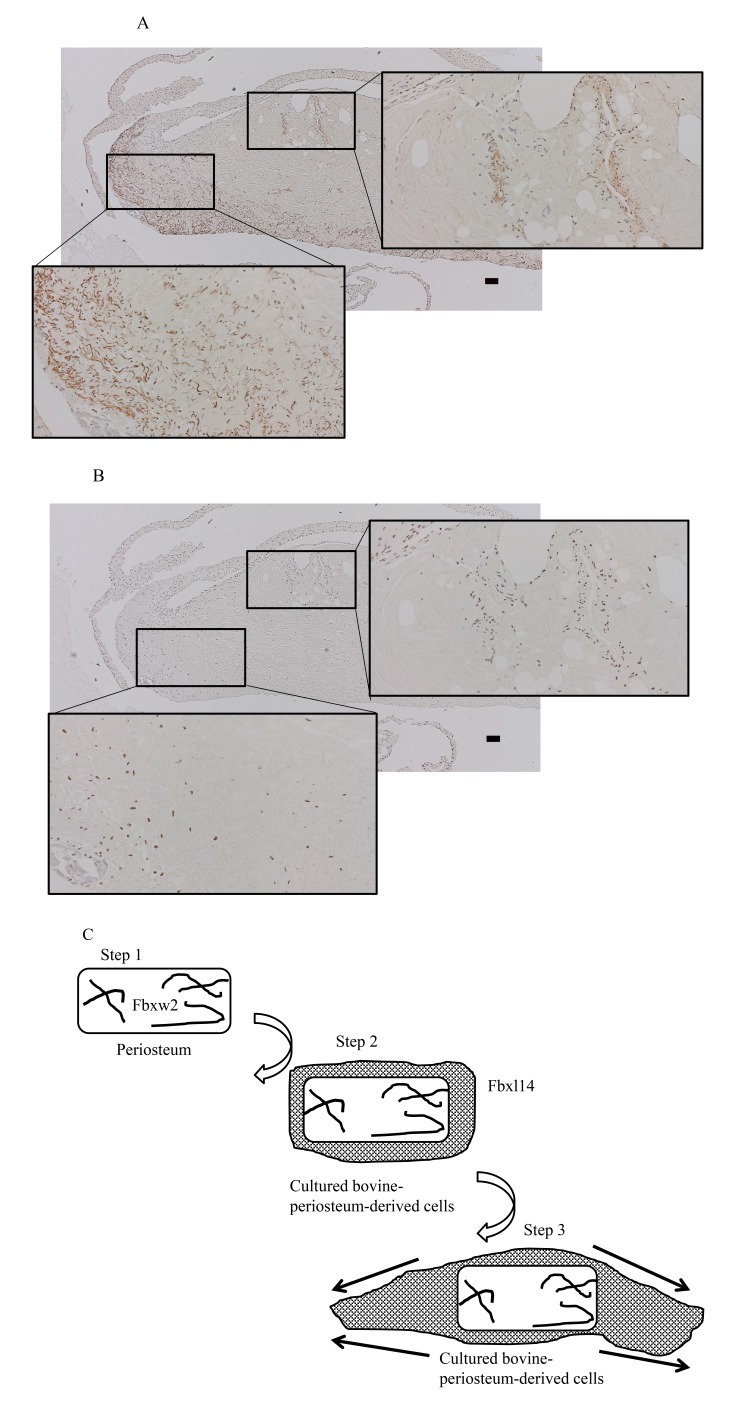
Hypothetical roles of the F-box proteins determined in this study. (**A, B**) Immunohistochemistry of FBXW2. Five weeks after explant culture of bovine periosteum. Scale bars: 100 µm. (**C**) Two F-box proteins, FBXW2 and FBXL14, are required for cell layer formation before bone formation.

Previously, Miura *et al*. [27] reported that the F-box protein FWD2 is predominantly expressed in the liver and is distributed in the cytoplasm. They suggested that SKP1, CUL1, and the F-box protein FWD2 form the ‘SCF’ complex, and that this complex plays a critical role in the ubiquitin-dependent degradation of proteins expressed in the liver. Hungwen Chen’s group also reported that the F-box protein FBW2 interacts with glial cell missing 1 (GCM1) and regulates the stability of GCM1 in the placenta [16, 20, 21, 28]. Similarly, Zheng *et al*. [29] reported that FBXL14 interacts with MKP3, which is expressed during axis formation in the zebrafish embryo. FBXL14 has also been reported to interact with and promote the ubiquitination of SNAL1, one of the SNAIL family of zinc-finger transcription factors [30]. The Human Protein Atlas website [31] presents a map of protein expression across 32 human tissues [32]. According to this map FBXW2 is strongly expressed in the small intestine and smooth muscle, whereas FBXL14 is expressed in the small intestine, endometrium, skin, tonsil, and 34 cell lines. However, the roles of FBXW2 and FBXl14 in the periosteum and bone are unclear. In the present study, two F-box proteins, FBXW2 and FBXL14, were detected in different sections: FBXW2 in the periosteum, but not in the cytoplasm, and FBXL14 in cultured periosteum-derived cells. This suggests that the roles of FBXW2 and FBXL14 differ. FBXW2 is only expressed in the periosteum in the bush-like tissue outside the cells, and not in the derived cells around the periosteum. Because FBXW2 is not present in cultured bovine-periosteum-derived cells, this protein might play a role in the transportation of primary cultured cells out of the firm collagen fibers of the periosteum (Fig. **8A**, **B**). Conversely, FBXL14 is present in the cultured cells around the periosteum. Therefore, FBXL14 might have a role in forming the cultured periosteum-derived cell layer around the periosteum and in cell migration (Fig. **8C**). Neither FBXW2 nor FBXL14 was expressed during bone formation, so these proteins might be required before bone formation. In summary, FBXW2 and FBXL14 are associated with cells that potentially form bone tissue, but their expression ceases in new bone tissue. The results of the present study show the changes in the expression of these proteins during the initiation stage, before bone formation.

In previous studies, cultured bovine-periosteum-derived cells regenerated bone after their transplantation into nude mice [10, 11]. For example, in 2006, Akiyama *et al*. [10] showed that osteocalcin was detectable with IHC 6 weeks after the transplantation of new bone tissue. In the present study, an anti-osteocalcin antibody was used as an osteoblast marker and TRAP staining was used as an osteoclast marker. HE staining and von Kossa staining revealed that osteocalcin was expressed around the new bone tissue and future bone tissue, whereas an IHC analysis of osteocalcin detected only osteoblasts in sections of transplanted cells, even though osteocalcin was strongly expressed in the study by Akiyama *et al*. [10]. One possible explanation for this discrepancy is that different osteocalcin antibodies targeting different regions of the protein were used in the two studies (present study: code no. M042, clone no. OCG2; previous study: code no. M041, clone no. OC4-30 [10]; Takara Bio Inc.). Different antibody dilutions were also used in the two studies. Specifically, in the present study, the osteocalcin antibody was diluted 1:500 to avoid background staining, whereas in the previous study, the antibody used was diluted 1:200 [10].

In the present study, I used cells at 4 weeks posttransplantation and a small area of new bone tissue to observe the process of bone formation (Fig. **5A**). At 4 weeks posttransplantation, the presence of osteoclasts, detected with TRAP staining, was remarkable because the cultured periosteum-derived cells caused bone formation after remodeling. Some periosteum-associated proteins have been widely used as markers of bone, including alkaline phosphatase, bone GLA protein, runt-related transcription factor 2 (RUNX2), osterix, and bone morphogenetic protein [2, 33]. However, the F-box proteins have not been reported to be associated with the periosteum or periosteum-derived cells. The present results explain this phenomenon by showing that the F-box proteins, such as FBXW2 and FBXL14, are present during the initiation phase of cell layer development, but are then lost during bone regeneration. Consistent with this, Akiyama *et al*. [10] demonstrated that the multiple layers of cultured bovine-periosteum-derived cells do not expand across the entire 100 mm of the tissue culture dishes, but expand radially for 15–20 mm around the periosteum in 100-mm tissue culture dishes containing ascorbic acid. Therefore, I hypothesize that cultured periosteum-derived cells are required to initiate the initial three-dimensional structure (Fig. **8C**). The model I propose is that in Step 1, FBXW2 is expressed in the periosteum. Subsequently (Step 2), cultured periosteum-derived cells expressing FBXL14 form around the periosteum cell layer. Finally (Step 3), the layer of cultured periosteum-derived cells becomes thick and moves to both sides.

## CONCLUSIONS

Bone regeneration requires progenitor cells, such as bovine-periosteum-derived cells and the activation of the F-box Proteins FBXW2 and FBXL14, over time the expression of these proteins ceases. Further scientific and clinical trials are needed to investigate how the F-box Proteins can be used therapeutically to treat osteoporosis and osteonecrosis.

## Figures and Tables

**Fig. (1):**
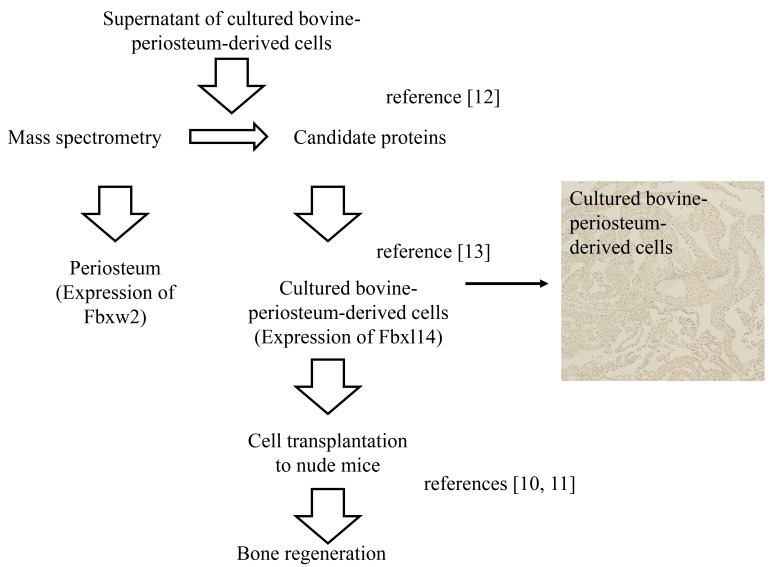
Outline of this study. A combination of MS and IHC identified periosteal-specific proteins. Periosteum, periosteal cells, cultured bovine-periosteum-derived cells, and new bone tissue generated with cell transplantation were examined.

**Fig. (2):**
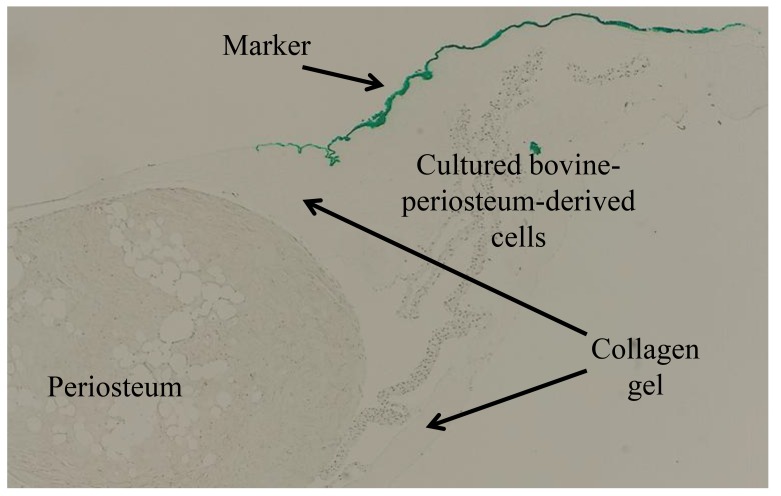
Outline of a cross-section of the periosteum and cultured bovine-periosteum-derived cells in collagen gel.

**Table 1:** Search parameters and the results of a MASCOT search for candidate protein FBXW2. Segments of the FBXW2 peptides detected in the supernatants of cultured bovine-periosteum-derived cells were matched.

Entry Name	FBXW2_BOVIN
Accession Number	Q58D00; Q58DP3
Full Name	F-box and WD-40 domain-containing protein 2
Database	SwissProt
Enzyme	Trypsin
Taxonomy	Other mammalia
Variable Modifications	Carbamidomethyl (C), Oxidation (M)
Peptide Mass Tolerance	50 ppm
Fragment Mass Tolerance	0.1 Da
Amino Acid Sequence	71–85: K.WLDPQTLLTCCLVSK.Q
Protein Sequence Coverage	3%

**Table 2:** Summary of the immunohistochemical analysis of FBXW2, FBXL14, and osteocalcin.

	Periosteum	Periosteal cells (in periosteum)	Cultured periosteum-derived cells (outside the periosteum)	Transplanted cultured periosteum-derived cells
FBXW2	○	×	×	×
FBXl14	×	○	○	×
Osteocalcin	×	○	○	○

## LIST ABBREVIATIONS

UACA: = Uveal Autoantigen with Coiled-coil domains and Ankyrin repeats
EXOSC9: = Exosome complex component RRP45
TMX2: = Thioredoxin-related transmembrane protein 2
FBXL14: = F-box/leucine-rich repeat protein 14
F-box/WD: = Repeat-containing protein 2 (FBXW2)
MS: = Mass Spectrometry ;IHC, Immunohistochemistry
TRAP: = Tartrate-Resistant Acid Phosphatase
FBS: = Fetal Bovine Serum
PFA: = Paraformaldehyde
HRP: = Horseradish Peroxidase
HE: = Hematoxylin and Eosin
